# Future research direction on decoding animal well-being: the case of the horse

**DOI:** 10.3389/fpsyg.2014.00173

**Published:** 2014-03-10

**Authors:** Nutankumar S. Thingujam

**Affiliations:** Department of Psychology, Sikkim UniversityGangtok, India

**Keywords:** animal wellbeing, observation, gender, age, personality, affectivity

The study by Lesimple and Hausberger (2014) compared in 26 cases the decoding of wellbeing of the horse by a caretaker through a questionnaire on the one hand and an experienced outside observer on the other hand. Stereotypic or abnormal repetitive behavior (SB/ARB) is what they study under the broad umbrella of wellbeing. They reported that there was a huge discrepancy in the prevalence of SB/ARB between the results obtained through the experienced observer (37% of the horses) and the caretakers' questionnaire responses (5%). On the basis of this result they conclude that there is underestimation in the judgment of wellbeing of the horses by the caretakers and they attribute it to the overexposure of the caretakers to their domestic horses.

I believe this is an interesting area of investigation and there is scope for further research. Among the several possible avenues of continued research one can examine if decoding of horse's SB/ARB is different across gender, age, affectivity (positive and negative), and personality dimensions of the observer because earlier work showed that judgment of human discrete emotions from the human face is influenced by gender (e.g., Hall and Matsumoto, 2004), age (e.g., Mill et al., 2009), affectivity (Rode et al., 2008), and personality (e.g., Matsumoto et al., 2000) of the decoder. But, such research attempt requires several observers to enable the use of inferential statistical analysis. For example, 30 male and 30 female trained observers could serve as the observers of the horse's SB/ARB with affectivity and personality measures as controlled variables. Alternatively, 30 young (say, 20–30 years) and 30 (say, 60 and above years) old trained observers could form the sample for a study in which affectivity and personality could be used as controlled variables.

## Observers' gender and decoding of animal wellbeing

In a study of human subjects Hall and Matsumoto (2004) found that women performed better than men in recognizing facial expressions of discrete emotions-anger, contempt, disgust, fear, and sadness. This finding suggests that human judgment of animal wellbeing in terms of horse's SB/ARB may also differ across the male and female trained observers (decoders).

## Age of the observer and decoding of animal wellbeing

In another study of human subjects it is reported that there is an age group difference in the recognition of discrete emotions of facial expressions- anger, contempt, disgust, fear, happiness, sadness, and surprise (Mill et al., 2009). Therefore, it is possible that there is an age group difference in human decoding (observation) of horse's well-being in terms of SB/ARB.

## Personality of the observer and decoding of animal wellbeing

Existing literature shows that personality of the observers (decoders) of human facial expressions of discrete emotions was associated with the higher accuracy of recognition of several emotional expressions. In particular, overall emotion recognition from the human face was associated positively with conscientiousness, openness to experience, extraversion, but negatively with neuroticism (Matsumoto et al., 2000). Similarly, other researchers also reported that human emotion decoding of sadness, happiness, and contempt from the facial expression was predicted by openness to experience; sadness decoding by agreeableness; anger recognition by extraversion (Mill et al., 2009). Hence, the relationship between human observer's personality and recognizing horse's wellbeing in terms of SB/ARB is still an important area to be investigated.

## Affectivity of the observer and decoding of animal wellbeing

As observers require several hours of observation one may speculate that the state and trait affect of the observer may influence the judgment of the animal's wellbeing. Past research has shown that human judgment of emotional expressions from the human face was associated with positive and negative affectivity (Rode et al., 2008).

The above suggestions could be applicable to the caretakers' judgment of animal wellbeing in terms of horse's SB/ARB. It is concluded that investigation of the influence of gender, age, personality, and affectivity of the observer as well as the caretaker is worth a look in human decoding of animal wellbeing in terms of SB/ARB.
